# Cancer classification in high dimensional microarray gene expressions by feature selection using eagle prey optimization

**DOI:** 10.3389/fgene.2025.1528810

**Published:** 2025-03-21

**Authors:** Swetha Dhamercherla, Damodar Reddy Edla, Suresh Dara

**Affiliations:** ^1^ Department of Computer Science and Engineering, National Institute of Technology, Farmagudi, Goa, India; ^2^ School of Computer Science and Engineering, VIT-AP University, Amaravati, Andhra Pradesh, India

**Keywords:** feature optimization, microarray gene selection, cancer classification, meta-heuristic optimization, feature selection

## Abstract

Microarray gene expression data have emerged as powerful tools in cancer classification and diagnosis. However, the high dimensionality of these datasets presents significant challenges for feature selection, leading to the development of various computational methods. In this paper, we utilized the Eagle Prey Optimization (EPO), a novel genetically inspired approach for microarray gene selection in cancer classification. EPO draws inspiration from the remarkable hunting strategies of eagles, which exhibit unparalleled precision and efficiency in capturing prey. Similarly, our algorithm aims to identify a small subset of informative genes that can discriminate between cancer subtypes with high accuracy and minimal redundancy. To achieve this, EPO employs a combination of genetic mutation operator with EPO fitness function, to evolve a population of potential gene subsets over multiple generations. The key innovation of EPO lies in its incorporation of a fitness function specifically designed for cancer classification tasks. This function considers not only the discriminative power of selected genes but also their diversity and redundancy, ensuring the creation of compact and informative gene subsets. Moreover, EPO incorporates a mechanism for adaptive mutation rates, allowing the algorithm to explore the search space efficiently. To validate the effectiveness of EPO, extensive experiments were conducted on several publicly available microarray datasets representing different cancer types. Comparative analysis with state-of-the-art gene selection algorithms demonstrates that EPO consistently outperforms these methods in terms of classification accuracy, dimensionality reduction, and robustness to noise.

## 1 Introduction

Cancer, one of the most formidable and devastating diseases afflicting humanity, is characterized by the uncontrolled growth and spread of abnormal cells within the body. Early and accurate diagnosis is paramount for effective treatment and improved patient outcomes. In the realm of cancer research, microarray gene expression profiling has emerged as a groundbreaking technology, offering insights into the intricate molecular mechanisms underlying various cancer subtypes. However, the high dimensionality of microarray data, characterized by thousands of gene features, poses a formidable challenge to the effective classification of cancer samples. In this context, the task of identifying a minimal subset of informative genes, while preserving the discriminative power of the data, becomes imperative. Gene selection, a critical preprocessing step in microarray analysis, aims to address this challenge by pinpointing the genes most relevant to the classification task. Numerous computational methods have been proposed to tackle this problem, ranging from filter-based approaches to wrapper-based algorithms. Nevertheless, the search for an optimal gene subset that maximizes classification accuracy and minimizes redundancy remains a complex and evolving area of research.

Whereas, the task of the feature selection is considered to be a fundamental and critical step in the process of data analysis, machine learning, and statistical modeling. It refers to the process of choosing a subset of relevant and informative features (or variables) from a larger pool of available features in a dataset. The primary objective of feature selection is to improve the performance of predictive models, reduce computational complexity, enhance interpretability, and mitigate the risk of overfitting. In real-world datasets, especially in fields such as biology, finance, image analysis, and natural language processing, it is common to encounter datasets with a multitude of features, some of which may be redundant, noisy, or irrelevant. The presence of such features can have adverse effects on the performance of machine learning algorithms. These effects include increased computational demands, reduced model generalization, and difficulty in understanding the underlying patterns in the data.

Feature selection offers a solution to these challenges by systematically identifying and retaining only those features that contribute the most to the predictive power of a model. By reducing the dimensionality of the data, feature selection simplifies the modeling process, often resulting in models that are easier to train, interpret, and deploy in practical applications.

Among the various available techniques for feature selection, the use of optimization techniques plays a pivotal role in feature selection. Feature selection involves choosing a subset of relevant features from a larger pool of variables to improve model performance, reduce computational complexity, and enhance interpretability. This process is essential for improving the efficiency and effectiveness of data analysis and modeling. Optimization methods offer a systematic and principled approach to tackling this challenging problem.

Firstly, optimization techniques provide a formal framework for defining and optimizing an objective function or criterion that quantifies the quality of a feature subset. The objective function can be tailored to the specific goals of feature selection, whether it is maximizing classification accuracy, minimizing computational cost, or achieving a trade-off between various performance metrics. Through this optimization process, one can systematically search through the space of all possible feature subsets to find the most promising subset that optimizes the chosen criterion.

Secondly, optimization methods enable the exploration of large and complex feature spaces efficiently. Given the exponential growth in the number of possible feature combinations with increasing data dimensionality, brute-force methods become computationally infeasible. Optimization algorithms, such as genetic algorithms, simulated annealing, or particle swarm optimization, offer efficient search strategies that can navigate through this vast feature space to identify relevant subsets, even in high-dimensional datasets.

Furthermore, optimization techniques allow for the incorporation of domain-specific knowledge and constraints into the feature selection process. Researchers and practitioners can encode their expertise or prior information into the optimization algorithm, ensuring that the selected feature subsets adhere to specific requirements or characteristics relevant to the problem domain.

Additionally, optimization-based feature selection methods facilitate the exploration of trade-offs between competing objectives. For instance, one might aim to simultaneously maximize classification accuracy while minimizing the number of selected features to reduce model complexity. Multi-objective optimization approaches can efficiently handle such scenarios, generating a range of Pareto-optimal solutions that represent the trade-off front between conflicting objectives.

Despite the advances in optimization algorithms for feature selection in microarray gene expression data, challenges remain in balancing exploration and exploitation to achieve efficient and effective gene selection. High-dimensional data, such as microarray data, poses inherent difficulties, including redundancy, noise, and the risk of overfitting in machine learning models. Existing meta-heuristic algorithms, while powerful, often suffer from premature convergence or excessive computational costs, especially when applied to datasets with high dimensionality and small sample sizes. This gap in achieving robust, efficient, and biologically interpretable solutions for cancer classification motivated the development of the Eagle Prey Optimization (EPO) algorithm. The inspiration behind EPO lies in the hunting strategies of eagles, which naturally balance global exploration and local exploitation.

In this context, Eagle Prey Optimization (EPO) emerges as a novel and genetically inspired approach designed to address the complex problem of microarray gene selection in cancer classification. EPO takes inspiration from the awe-inspiring hunting strategies of eagles, which are renowned for their precision and efficiency in capturing prey. In a similar vein, EPO seeks to identify a concise and informative set of genes that can effectively discriminate between different cancer subtypes while minimizing redundancy and maintaining robustness. This paper introduces the concept of Eagle Prey Optimization and presents its application in the domain of microarray gene selection for cancer classification. Through rigorous experimentation and comparative analysis with state-of-the-art algorithms, we demonstrate the superior performance of EPO in terms of classification accuracy, dimensionality reduction, and robustness to noise. Furthermore, we highlight the algorithm’s ability to unveil biologically relevant genes associated with cancer pathways, thereby contributing to our understanding of the molecular basis of cancer subtypes. The integration of EPO in cancer research not only enhances the diagnostic potential but also holds the promise of discovering novel biomarkers and therapeutic targets, ultimately advancing the field of precision medicine and improving the prognosis of cancer patients. This paper delves into the intricacies of Eagle Prey Optimization, its genetic-inspired mechanisms, and its potential to revolutionize microarray gene selection in cancer classification.

This paper presents several significant contributions that advance the field of cancer classification and microarray gene selection using the proposed EPO algorithm. The main contributions of the work are given as:

• Introduces Eagle Prey Optimization, a novel optimization algorithm inspired by the hunting strategies of eagles. EPO is specifically tailored for microarray gene selection in cancer classification, offering a unique approach to address the challenges associated with high-dimensional gene expression data.
• EPO leverages genetic-inspired mechanisms to evolve a population of potential gene subsets over multiple generations. This genetic inspiration allows EPO to explore the search space effectively and efficiently, mimicking the precision and efficiency of eagles in hunting prey.
• EPO incorporates a specialized fitness function designed for cancer classification tasks. This function takes into account not only the discriminative power of selected genes but also their diversity and redundancy, promoting the creation of compact and informative gene subsets that enhance classification accuracy.
• Provides comprehensive experimental results of the EPO using the various different cancer type datasets having high dimensional feature representation and comparing EPO with state-of-the-art gene selection algorithms.

The rest of the paper is organized as follows: Section 2 provides an overview of the existing literature on feature selection methods for microarray data in cancer classification. It discusses various optimization algorithms, genetic-inspired approaches, and their applications in gene selection. The paper delves into the details of the proposed EPO algorithm in Section 3. It explains the genetic-inspired mechanisms used in EPO and the dedicated fitness function for cancer classification is described in depth, highlighting its role in promoting the selection of informative and non-redundant gene subsets. The experimental methodology is presented in Section 4. It outlines the datasets used for validation, the performance metrics employed, and the results of the experiments conducted to evaluate the performance of EPO. The paper concludes by summarizing the key findings and contributions of the research in Section 6.

## 2 Literature review

Microarray gene expression data analysis has significantly advanced our understanding of cancer biology and has played a pivotal role in cancer classification and diagnosis. However, the high dimensionality of microarray data, characterized by thousands of gene expression profiles, poses a major challenge. Feature selection techniques have become essential in addressing this challenge by identifying a subset of informative genes that are most relevant for cancer classification. In the past literature on microarray gene expression feature selection, extensive research has been conducted to develop and evaluate various methods and techniques. These methods aim to identify a subset of relevant genes from the large pool of gene expression data to improve the accuracy and interpretability of cancer classification models.

On a similar theme, the work introduced in (Peng et al., 2005), proposed a feature selection method that maximizes mutual information while minimizing redundancy among selected features, enhancing the informativeness of the selected genes for cancer classification. Whereas, a team of several authors also worked on the comparative analysis of feature selection (Almugren and Alshamlan, 2019; Mohd Ali et al., 2022; Remeseiro and Bolon-Canedo, 2019; Alhenawi et al., 2022). These studies evaluate various feature selection methods and multiclass classification algorithms for microarray-based tissue classification. They provide insights into the best approaches for accurate cancer subtype classification.

The work proposed in (Houssein et al., 2022), presents an integrated approach combining manta rays foraging optimization and support vector machine methods for gene selection and cancer classification, achieving improved classification performance. Using the strategy of optimization only, the work proposed in Othman et al. (2020) uses the gene selection algorithm inspired by the cuckoo search optimization algorithm with evolutionary operators for cancer microarray data. It demonstrates improved performance in terms of classification accuracy and feature selection efficiency. Also, the authors of Aziz (2022) use the hybrid approach, using cuckoo optimization as one algorithm for cancer classification. The use of the cuckoo search optimization algorithm is also found satisfactory in collaboration with other optimization algorithms, making a hybrid approach for various application areas (Priya et al., 2022; Senapati et al., 2023; Kalaiarasu and Anitha, 2020; Segera, 2021).

Several authors also showed the reliability of the genetic algorithm on microarray gene expression datasets for feature reduction and dimensionality reduction. One such work is proposed in Ram and Kuila (2019) where the authors present a genetic algorithm for microarray gene selection. It employs a feature ranking strategy to improve classification accuracy and reduce dimensionality. In addition to the genetic algorithms, the use of manifold learning is also utilized by some authors for cancer classification using the gene dataset (Wang et al., 2023). In a couple of recent works, the genetic algorithm is also used in the hybridization of the algorithms for the selection of the more robust features for high-dimensional cancer datasets (Ge et al., 2019; Ali and Saeed, 2023). The results from these research articles boost the interest in using hybrid algorithms as they improve classification accuracy. Besides this, the use of genetic algorithms is also found to enhance the effectiveness of similarity searching in ligand-based virtual screening which is proposed in Berrhail and Belhadef (2020). Some of the authors explored the use of the feature-thresholds guided genetic algorithm for feature scoring on high-dimensional datasets (Deng et al., 2023). This new variant of the genetic algorithm improves the classification accuracy by using a limited set of selected genes.

In one of the works on high-dimensional datasets, the literature introduces a distance ratio-based feature selection algorithm that considers both inter-class and intra-class distances to identify informative genes for cancer classification (Brankovic et al., 2018). Similarly in the article (Zhou et al., 2022), authors proposed a new approach using the mutual information with correlation coefficient for feature selection on high dimensional datasets. Whereas, the critical analysis of the various feature selection approaches and their stability prediction is presented in one of the current research studies (Khaire and Dhanalakshmi, 2022).

In some of the works, the authors proposed the use of the quantum approach with the optimization algorithms for reducing high dimensional data. On this, the work introduces a quantum binary particle swarm optimization algorithm tailored for feature selection in gene expression data, highlighting its ability to identify relevant genes for cancer classification with high efficiency (Wu et al., 2019). Similarly, in Ghosh et al. (2021) the authors proposed a quantum squirrel-inspired algorithm for gene selection in methylation and expression data of prostate cancer. The work claimed that the quantum-inspired algorithm variant provides good results with the selection of the relevant genes. Several other variants of the quantum-inspired form of various optimization algorithms were also present in the literature for the selection of the relevant genes from the high dimensional dataset (Dabba et al., 2020; Wang et al., 2020). Also, the details of the quantum meta-heuristic algorithms and their role in various engineering applications were also presented in the literature (Prakash, 2021).

In some of the recently published works on gene selection for cancer classification, authors have used a variety of approaches. One such approach is the use of hybridization. In Mahesh et al. (2024), authors have proposed the use of the hybrid model that integrates the strengths of Ant Colony Optimization (ACO) and Particle Swarm Optimization (PSO) to enhance feature selection and classification accuracy. The model’s effectiveness is demonstrated through its application in predicting leukemia, achieving a notable accuracy of 87.88% using a Support Vector Machine (SVM) for classification. In another recent work, proposed in 2024 (Sucharita et al., 2024), authors proposed a two-step hybrid approach that begins with an ensemble of filter-based heterogeneous feature selection methods. This initial step is crucial as filter methods are known for their efficiency in handling high-dimensional datasets by evaluating the relevance of features based on statistical measures without involving any classifier. In the second step, the selected features undergo a wrapper-based selection process. Here, the authors employ the Moth-Flame Optimization (MFO) algorithm, which is a bio-inspired optimization technique (Zamani et al., 2024). The fitness function for this optimization is based on an Extreme Learning Machine (ELM), which is known for its rapid learning capabilities and one-pass processing of samples. This characteristic of ELM allows for efficient training, making it suitable for scenarios where computational resources are limited.

In another work, an author used the two computational approaches for cell-type shared and specific binding (Zhang et al., 2023). Authors characterize cell-type-specific and shared binding sites by integrating multiple types of features, utilizing XGBoost and convolutional neural networks (CNNs). The integration of diverse features is found crucial in their study for enhancing model performance in biological contexts as evidenced by recent studies that highlight the importance of feature diversity in improving predictive accuracy for transcription factor (TF) binding sites. The experimental results demonstrate that both the XGBoost and CNN models significantly outperform existing methods across three classification tasks, supporting the assertion that advanced machine-learning techniques can effectively capture the complexities of biological data.

Besides the microarray gene expression data, there exists work in the literature on another class of dataset called next-generation sequencing (NGS) data. Optimization techniques can be applied to next-generation sequencing (NGS) data in various ways to extract valuable insights and enhance the analysis of biological and genomic information. NGS data optimization typically focuses on improving data quality, computational efficiency, and the extraction of biologically relevant information. Many authors worked on this domain recently to optimize the NGS data using optimization approaches.

One such different work is proposed in McNulty et al. (2019) on the NGS dataset. This research contributes to the field of cancer genomics by offering a data-driven and adaptable solution for filtering common germline polymorphisms from tumor-only NGS data. The optimized cutoffs provide a more reliable basis for somatic mutation identification, improving the precision and clinical relevance of cancer genomics research and personalized medicine.

In the work (Pellegrino et al., 2023), the authors used the fusion of Extreme Gradient Boosting and metaheuristic algorithms to provide a robust and effective framework for predicting pathogenicity in myeloid NGS onco-somatic variants. The integration of these techniques holds the potential to enhance our understanding of the genetic underpinnings of myeloid malignancies, supporting precision medicine initiatives and improving patient outcomes in the field of onco-hematology.

The work proposed by the authors of Halim (2020) considered the optimization of the DNA fragment assembly as a critical task in genomics and bioinformatics, where researchers aim to reconstruct the complete DNA sequence from a set of smaller overlapping fragments. The Overlap-Layout-Consensus (OLC) approach here is a fundamental method in this process, and metaheuristic-based techniques offer innovative ways to enhance the efficiency and accuracy of DNA fragment assembly. Whereas in MotieGhader et al. (2020), the research contributes to the advancement of breast cancer molecular subtype stratification by introducing a novel approach that integrates meta-heuristic optimization algorithms with feature selection criteria for mRNA and microRNA data. The results demonstrate the efficacy of this approach in enhancing classification accuracy and illuminating the biological underpinnings of breast cancer subtypes, ultimately paving the way for personalized treatment strategies.

## 3 Proposed eagle prey optimization for feature subset selection

Eagle prey hunting is a remarkable and highly efficient hunting strategy employed by various species of eagles, which are large and powerful birds of prey known for their exceptional hunting abilities. Eagles possess exceptional eyesight, with some species capable of spotting prey from great distances. Their acute vision allows them to identify potential targets with remarkable precision. Eagles often begin their hunts by soaring at high altitudes, scanning the ground below for potential prey. Their ability to see over large areas from a great height gives them a strategic advantage. Once a suitable target is spotted, eagles use their impressive speed and maneuverability to initiate a surprise attack. They typically approach their prey from above, diving down to catch their target off guard. Eagles have powerful talons with sharp claws designed to grip and immobilize their prey effectively. The talons are used to secure the prey and prevent it from escaping. They can adjust their trajectory and speed during the hunt to ensure a successful capture. Whereas, the choice of prey can vary from small mammals, birds, and fish, to even larger prey.

Eagles employ a variety of hunting techniques depending on their species and the type of prey they are targeting. The hunting strategy of the eagles is known with some common situational conditions like:

• *Aerial Hunting*: Many eagle species are skilled aerial hunters. They soar at high altitudes, scanning the ground for potential prey. When a suitable target is spotted, the eagle dives down with incredible speed and accuracy to seize its prey. This technique is often used for hunting birds, which are captured mid-air.
• *Perch and Wait:* Some eagles, such as the African fish eagle, prefer to perch near water bodies. They wait patiently for fish or other aquatic prey to come close to the surface, then swoop down to capture them with their sharp talons.
• *Surprise Attacks:* Eagles are known for their ability to launch surprise attacks on their prey. They approach from behind obstacles or use the sun to blind their prey, making it harder for the prey to detect them until it is too late.
• *Cooperative Hunting:* Eagles engage in cooperative hunting, particularly when targeting larger prey. Bald eagles, for instance, may work together to capture larger fish. One eagle distracts the prey while the other swoops in for the capture.
• *Still Hunting:* Some eagle species, like the martial eagle, are known for “still hunting.” They perch inconspicuously in trees or on cliffs, silently waiting for ground-dwelling prey to come into their field of view. When the prey is within striking distance, the eagle pounces with great force.
• *Scavenging:* While eagles are primarily hunters, they are opportunistic and sometimes scavenge for carrion or steal food from other birds or predators. Bald eagles, for example, are known to scavenge fish from other birds or steal from otters.
• *Hunting Grounds:* Eagles tend to establish hunting grounds in their territories, often returning to the same locations repeatedly. They become familiar with the behavior of prey in their territory, improving their hunting success.
• *Stalking and Ambushing:* Some eagles stalk their prey on the ground, using cover and vegetation to approach undetected. Once in range, they swiftly ambush and capture their prey.

**FIGURE 1:**
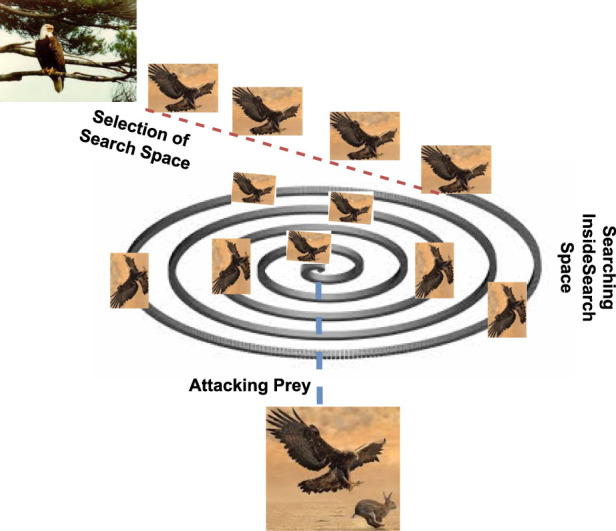
Prey hunting strategy of the eagle including the selection of the search space, searching prey in search space and executing an attack.

Based on the above-defined approaches, the eagle hunting strategies can indeed be summarized in a sequence of actions: that includes selecting the search space, searching for prey within that space, and executing an attack. This behavior of the eagle targeting the prey is shown diagrammatically in Figure 1.

### 3.1 Selection of the search space

The eagles choose a certain region to start their quest for food and depart in a specified direction once they have settled on a target area. As a result, discovering the search space is accomplished through self-searching and tracking other birds. Eagles often begin their hunting expedition by ascending to a significant altitude, sometimes thousands of feet in the air. This altitude provides them with a broader view of the landscape and potential prey. The search space encompasses the area below the eagle, covering a wide territory. It is from this vantage point that eagles identify potential targets.

### 3.2 Searching inside the search space

After selecting an appropriate search space, the eagles will start to search for the prey in the search space. Eagles have exceptional eyesight, and they scan the terrain for any signs of movement or potential prey items. Their keen vision enables them to detect even small or distant prey. They focus on specific characteristics, such as the size, shape, or behavior of animals on the ground and any movement that may indicate the presence of prey. The search often involves circling or gliding at various altitudes to cover a larger area effectively. Generally, the searching behaviors of the eagles are in spiral form with the selected search space.

After the scanning of the prey from the specific altitude and once a suitable prey item is identified within the search space, the eagle initiates its attack. The decision to attack is based on factors like the proximity and vulnerability of the prey. Eagles are known for their remarkable speed and agility during the attack phase. They may adjust their altitude, speed, and direction as needed to maintain the element of surprise and increase the accuracy of their strike. The attack typically involves a rapid descent, with the eagle using its sharp talons to grasp and immobilize the prey. The talons are designed to pierce and grip the prey securely, ensuring that it cannot escape.

### 3.3 Proposed eagle prey optimization design framework

Any nature-inspired meta-heuristic algorithm starts with the random initialization of the seed point. This seed point acts as a base parameter to start with and the corresponding objective function either needs to be minimized or maximized depending on the choice of the problem. Let us suppose that the sample space is defined using the 2-dimensional space of size *m* × *n* and is given by Equation 1.

$$A=\left[\begin{array}{c} A_{1}\\ A_{2}\\ \ldots \\ A_{i}\\ \ldots \\ A_{n} \end{array}\right]=\left[\begin{array}{cccccc} A_{1}^{1} & A_{1}^{2} & \ldots & A_{1}^{j} & \ldots & A_{1}^{m}\\ A_{2}^{1} & A_{2}^{2} & \ldots & A_{2}^{j} & \ldots & A_{2}^{m}\\ \ldots & \ldots & \ldots & \ldots & \ldots & \ldots \\ A_{i}^{1} & A_{i}^{2} & \ldots & A_{i}^{j} & \ldots & A_{i}^{m}\\ \ldots & \ldots & \ldots & \ldots & \ldots & \ldots \\ A_{n}^{1} & A_{n}^{2} & \ldots & A_{n}^{j} & \ldots & A_{n}^{m} \end{array}\right]$$ (1)

where the symbolic representation of the used variables is defined as the total number of sample objects given by variable n, the problem dimension is represented by m, $A_{i}^{j}$ is deciding the beginning position of the $i^{th}$ candidate for $j^{th}$ decision variable.

In this sample space, the Eagle will select the hunting space heuristically and identify the region having the maximum possible chance of the prey, called the best position for hunting, called $Pos_{\mathrm{best}}$ . The next iterative position for hunting the prey is given by Equation 2.

$$Pos_{i,j}=Pos_{\mathrm{best}}+\alpha \times \beta \times \left(Pos_{\mathrm{mean}}-Pos_{i}\right)$$ (2)

where, α and β are the exploding learning factor and selected random number respectively in the range of (0, 1).

The process starts with random solutions of candidate solution as $\left(Pos_{ij}\right)$ in the range between $Pos_{\mathrm{mean}}$ and lower limits $\left(Pos_{i}\right)$ , where $i^{th}$ is the problem dimension.

Next, the second step of hunting is the searching of the prey in the selected region. As the eagle searches for its prey in the spiral form and thus at every temporal state this diameter of the spiral reduces by a factor till it swoops the prey. This process is mathematically given by Equation 3.

$$Pos_{1}\left(t+1\right)=Pos_{\mathrm{best}}\left(t\right)\times \gamma +\left(Pos_{\mathrm{mean}}\left(t\right)-Pos_{\mathrm{best}}\left(t\right)\times \beta \right)$$ (3)

where, γ is the exploration parameter in range (0, 1), $Pos_{1}\left(t+1\right)$ is eagle position in the next iteration *t*, $Pos_{\mathrm{best}}\left(t\right)$ is the best possible solution till $t^{th}$ timestamp and $Pos_{\mathrm{mean}}$ is the mean value of the best position values till timestamp *t*.

Once the target is found, the eagle will shorten the spiral circle radius and focus on the prey to swoop. This process is known as narrowing exploration in the search space and mathematically is given by Equation 4.

$$Pos_{2}\left(t+1\right)=Pos_{\mathrm{best}}\left(t\right)\times F\left(D\right)+Pos_{\mathrm{rand}}\left(t\right)+\left(Y-X\right)\times \left.\beta \right)$$ (4)

where $Pos_{\mathrm{rand}}\left(t\right)$ is the random solution at time t using the $i^{th}$ iteration, F(D) is the flight distribution function in D dimension space and is given by Equation 5.

$$F\left(D\right)=0.01\times \frac{u\times \omega }{|v{|}^{1/b}}$$ (5)

Here u and v are selected randomly in the range of (0, 1), b is constant having a value of 1.5, and ω is an adaptive parameter with the value calculated using Equation 6.

$$\omega =\frac{b\times sin\left(\frac{\pi \times b}{2}\right)}{b^{2}\times 2^{\left(\frac{b-1}{2}\right)}}$$ (6)

Also, the Y and X are the coefficients that handle the spiral space of the eagle moment. The mathematical formulation of these is given in Equation 7.

$$X=\frac{xr\left(i\right)}{argmax\left(\left|xr\right|\right)},\;Y=\frac{yr\left(i\right)}{argmax\left(\left|yr\right|\right)}$$ (7)

where r(i) is given as,

$$r\left(i\right)=\Theta \left(i\right)+SC\times \beta ;\;\;\Theta \left(i\right)=a\times \pi \times \beta$$

Here, SC is the search cycle and holds a value in the range (0,5), and a is the rotation parameter in the range (2,10). Thus the parameters a and SC were used to handle the change in the spiral shape.

The next hunting phase of the eagle is called the exploitation phase where the eagle swoops the prey with a slow encounter which is mathematically given by Equation 8.

$$Pos_{3}\left(t+1\right)=0.1\times \left(Pos_{\mathrm{best}}\left(t\right)-Pos_{\mathrm{mean}}\left(t\right)\right)-0.2\times \left(\beta \times \left(Pos_{\mathrm{mean}}-Pos_{i}\right)+Pos_{i}\right)$$ (8)

Here, the constants 0.1 and 0.2 are the adjustment parameters of the exploitation phase and are fixed using simulation results.

The other case is when the prey is large enough to swoop on the ground, Equation 8 is to be narrowed down as the hunt is more forced and speedy. The modified equation of the narrow exploitation phase is given by Equation 9.

$$Pos_{4}\left(t+1\right)=\left(t^{\frac{2\beta -1}{{\left(1-T\right)}^{2}}}\times Pos_{\mathrm{best}}\left(t\right)\right)-\left(2\beta \times Pos\left(t\right)\right)-\left(2\gamma \times F\left(D\right)\right)+2\beta$$ (9)

Here (1-T) represents the amount of time the eagle spent to swoop the prey from the ground from the total time spent on hunting.

**FIGURE 2:**
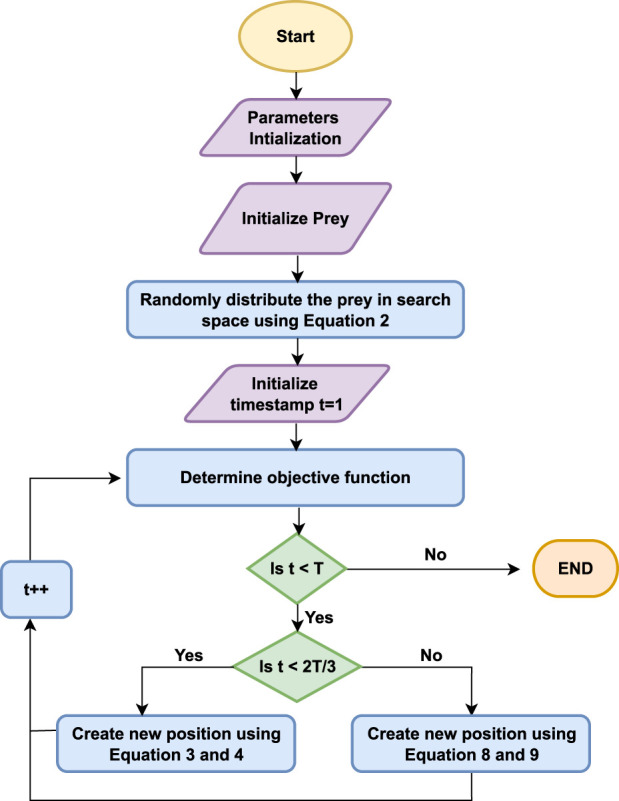
Flowchart of the proposed Eagle Prey Optimization algorithm.

The stepwise explanation of the proposed algorithm is shown using the flowchart in Figure 2. Whereas, the pseudo-code of the proposed EPO algorithm is given in Algorithm 1.

### 3.4 Hyperparameter selection and tuning

The initial ranges of hyperparameters were selected based on prior literature and the nature of the optimization problem. The population size $\left(N_{p}\right)$ is set to 100, as smaller populations are computationally efficient, while larger populations improve solution diversity. The exploration factor (w) is initialized between 0.4 and 0.9 to balance exploration and exploitation. While the cognitive and social components ( $C_{1}$ and $C_{2}$ ) both set in the range of 1.5–2.5. These parameters are the common practice in meta-heuristic optimization algorithms. Also, we conducted a series of small-scale preliminary experiments to assess the sensitivity of EPO’s performance to hyperparameter values. These experiments were performed on a subset of the dataset using a fixed number of iterations to reduce computational overhead.

A grid search was performed to fine-tune critical hyperparameters ( $N_{p},w,c_{1}$ , and $c_{2}$ ). Each combination was evaluated using a fitness function based on classification accuracy achieved by the selected gene subset. During training, we implemented an adaptive strategy for the exploration factor (w) to dynamically shift from exploration to exploitation as given by the following Equation 10.

$$W=W_{\max}-\frac{\left(W_{\max}-Wmin\right)\times t}{T}$$ (10)

where $W_{\max}$ and $W_{\min}$ are the initial and final values of W , t is the current iteration, and T is the total number of iterations. After identifying promising hyperparameter combinations, further fine-tuning was performed based on the validation accuracy of the five machine learning classifiers used in the study.

### 3.5 Fitness calculation

The fitness function f in the proposed Eagle Prey Optimization algorithm evaluates the quality of each candidate solution (subset of selected genes) by balancing two critical objectives i.e., *Classification performance* and *minimization of feature count*. The primary goal of the fitness function is to maximize the classification accuracy of a machine learning model trained on the selected gene subset. The classification performance is quantified using performance metrics. For our experiments, we primarily use accuracy as a proxy for classification performance. Also, to address the high dimensionality of microarray data, the fitness function incorporates a penalty term that discourages the selection of large gene subsets. This promotes compact and computationally efficient feature subsets. The designed fitness function for EPO algorithm covering both objectives is given by Equation 11.

$$f\left(s\right)=\alpha .A+\left(1-\alpha \right).\left(1-\frac{N_{s}}{N_{t}}\right)-W.P\left(s\right)$$ (11)

where A is the accuracy, $N_{s}$ is the number of selected features (genes), $N_{t}$ is the total number of features (genes) in the dataset, α is the weight parameter (0≤α≤1) that controls the trade-off between classification performance and feature subset size, s is the selected feature set, W is the weight coefficient that balance the importance of redundancy, and P(s) penalizes highly correlated features.

Algorithm 1. An algorithm of Proposed Eagle Prey Optimization.

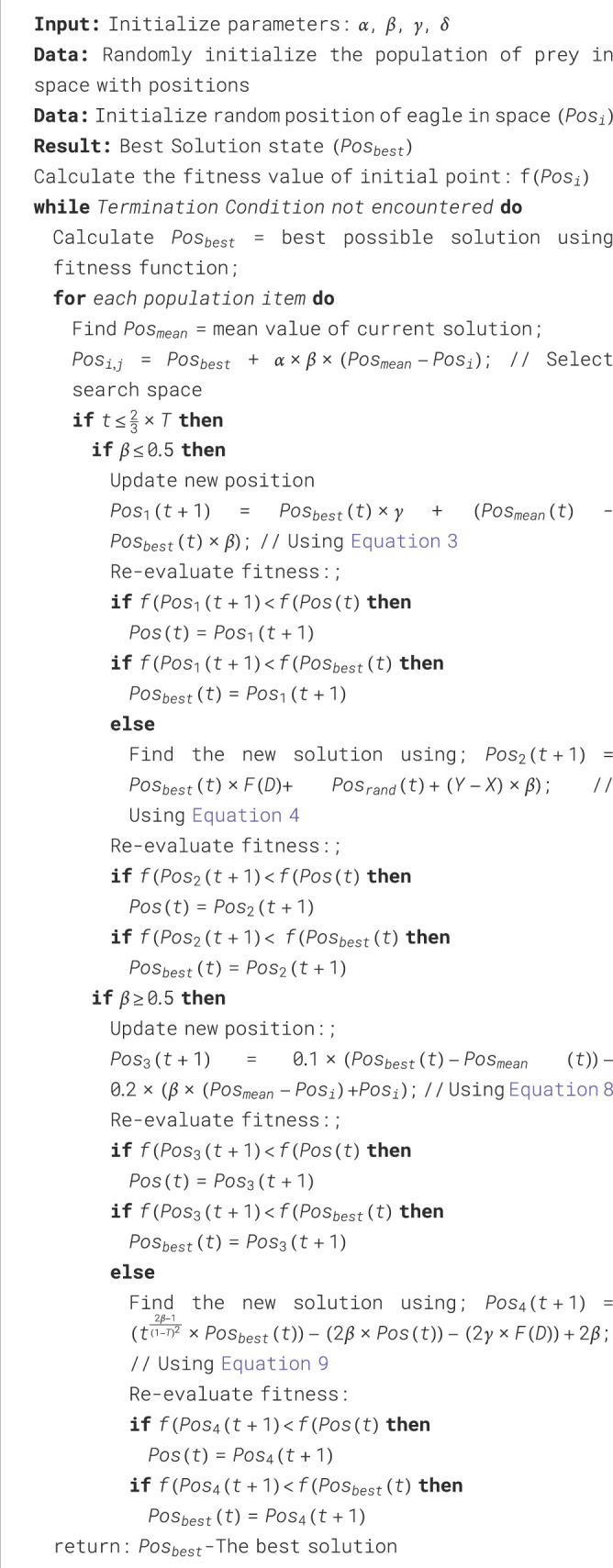

### 3.6 Complexity analysis

The computational complexity of the EPO algorithm is determined by three primary components.

1. Initialization Phase: The algorithm starts with the initialization of a population of candidate solutions (prey) of size N . Each candidate solution represents a subset of selected features. The cost of initializing each candidate solution is proportional to the total number of features $\left(N_{t}\right)$ . Thus the complexity is $O\left(N.N_{t}\right)$ .
2. Fitness Evaluation: The fitness of each candidate solution is computed using the defined fitness function, which includes evaluating a machine learning model on the subset of features, and the cost of training and validating the machine learning model depends on the size of the selected subset $\left(N_{s}\right)$ and the number of training samples (M) .

For a single fitness evaluation the complexity is $O\left(N_{s}.M\right)$ and for all candidates in the population the complexity is $O\left(N.N_{s}.M\right)$ .

3. Exploration and Exploitation Phases: In each iteration, the algorithm performs exploration and exploitation to update the positions of the candidate solutions. This involves calculating the movement of prey in the search space based on adaptive mechanisms and updating positions and recalculating fitness for N candidate solutions. The cost of these operations is proportional to the population size N and the dimensionality of the problem $N_{t}$ . Thus the complexity per iterations is $O\left(N.N_{t}\right)$ .

The algorithm performs these operations for a total of I iterations, where I is the maximum number of iterations or until convergence. This will give the complexity over all iterations as $O\left(I.N.N_{t}\right)$ . Combining the components above, the overall computational complexity of the EPO algorithm is $O\left(I.N.\left(N_{t}+N_{s}.M\right)\right)$ .

## 4 Experimentation results

### 4.1 Dataset description

To evaluate the performance of the proposed feature selection method, in the experimentation, the Microarray Gene Expression (MGE) dataset is taken into consideration. The dataset is available on the public data repository^1^ having 11 variants of the cancer genes supervised into binary to multi classes. Out of the 11 available gene expression datasets, we took 8 datasets (except ALL-AML, ALL-AML-3, and ALL-AML-4). The selected 8 datasets are in the. arff extension having gene counts, instances count, and number of class distribution information. The choice of the selection of this data repository is because of having the minimum gene count of 2000, which is a pre-screened criterion of dataset selection. The description of the dataset with the parameter information is presented in Table 1.

**TABLE 1:** Dataset Description with the associated parameter information.

Datasets	#Gene	#Instance	#Class
Colon Tumor	2000	60	2
Central Nervous System	7129	60	2
Breast Cancer	24,481	97	2
Lung Cancer	12,533	181	2
Ovarian Cancer	15,154	253	2
Lymphoma	4026	62	3
MLL	12,582	72	3
SRBCT	2308	83	4

### 4.2 Dataset splitting

In the machine learning system, while model training, the common practice is to subdivide the dataset into two parts i.e., Training and validation. To divide the dataset, the most commonly used approach is the 80-20 rule, where the randomly selected 80% dataset is used as a training set and the rest 20% as validation. But, with the smaller dataset having a limited sample size this approach is found appropriate, more especially with the MGE datasets as reported in Braga-Neto and Dougherty (2004). Thus, in this work, the dataset has experimented with the well-known Bootstrap method, called the 0.632+ estimator (Efron and Tibshirani, 1997).

#### 4.2.1 Compute error loss with cross-validation approach

Let’s say we wish to use the function f to forecast V using U such that f may depend on some parameters that are predicted from the data (V, U), i.e., f(U) = α× U. The error estimate of this sample is calculated using Equation 12.

$$error\left(\hat{e}\right)=\frac{1}{N}\overset{N}{\underset{i=1}{\sum }}\hat{L}\left(v_{i},f\left(u_{i}\right)\right)$$ (12)

where $\hat{L}$ is the mean square error loss function in model training.

As the function f tried to fix the data using the given sample $\left(u_{i},v_{i}\right)$ only, the chance of having a negative bias can be formed. To reduce this the approach of k-fold cross-validation can be utilized. In the K-fold CV approach, we divide our dataset into K subgroups say 5, for example,. Fit your model on the remaining K-1 subgroups for each group k, then test it on the $k^{th}$ group. The Error equation using k-fold is updated as given in Equation 13.

$$error_{k_{\mathrm{fold}}}\left({\hat{e}}_{k_{\mathrm{fold}}}\right)=\frac{1}{N}\overset{N}{\underset{i=1}{\sum }}\hat{L}\left(v_{i},f_{-k\left(i\right)}\left(u_{i}\right)\right)$$ (13)

where $f_{-k\left(i\right)}\left(u_{i}\right)$ is the anticipated value of $u_{i}$ using data not in the $k{\left(i\right)}^{th}$ set and k is some index function that identifies the partition to which observation i is allocated.

When using cross-validation, the value of k, or the number of folds to utilize, is crucial. The error estimates have an increase in the bias and lower variance with the decreasing value of k. On the other hand, the error estimate may have a significant variance but a very low bias when k is set to the number of instances. Once more, the bias-variance tradeoff may result from this. In such a condition we prefer the use of bootstrapping over cross-validation.

#### 4.2.2 Computing error loss with 0.632+ estimator

To estimate the extra-sample prediction error, we might utilize the bootstrap method rather than cross-validation. Any statistic’s sample distribution may be estimated via bootstrap resampling. We can consider picking *B* bootstrap samples (with replacement) from the set $S_{1},\ldots ,S_{B}$ , where each $S_{i}$ represents a set of *N* samples, if our training data is X = $\left(u_{1},\ldots ,u_{N}\right)$ . We can now estimate the extra sample prediction error using leave-one-out bootstrap samples as given in Equation 14.

$$error_{B}\left({\hat{e}}_{B}\right)=\frac{1}{N}\overset{N}{\underset{i=1}{\sum }}\frac{1}{\left|C^{-i}\right|}\underset{b\in C^{-i}}{\sum }\hat{L}\left(v_{i},f_{b}\left(u_{i}\right)\right)$$ (14)

where $f_{b}\left(u_{i}\right)$ is the predicted value at $u_{i}$ from the model fit to the $b^{th}$ bootstrap dataset, $C^{-i}$ is the set of indices for the bootstrap samples that do not contain observation i, and $\left|C^{-i}\right|$ is the number of such samples.

The above Equation 14 addresses the overfitting issue, but retains bias. The non-distinct observations in the bootstrap samples, a consequence of replacement sampling, are the cause of this bias. Thus, for every sample size, the average number of unique observations can be around 0.632N (Efron and Tibshirani, 1997). Thus, the error computation using the 0.632+ estimator is given by Equation 15.

$$error_{0.632+}=\left(1-w\right)\times \bar{error}+w\times error_{B}$$ (15)

where,

$$\bar{error}=\frac{1}{N}\overset{N}{\underset{i=1}{\sum }}\hat{L}\left(v_{i},f\left(u_{i}\right)\right),\;\;\;\;\;\;w=\frac{0.632}{1-0.368R}$$

and

$$R=\frac{error_{B}-\bar{error}}{\Gamma -\bar{error}},\;\;\;\;\;\;\Gamma =\frac{1}{N^{2}}\overset{N}{\underset{i=1}{\sum }}\overset{N}{\underset{j=1}{\sum }}\hat{L}\left(v_{i},f\left(u_{j}\right)\right)$$

Here, R measures the relative overfitting rate and Γ is the no-information error rate, estimated by evaluating the prediction model on all possible combinations of targets $v_{i}$ and predictors $u_{i}$ .

### 4.3 Performance measurement parameters

Performance evaluation and goodness-of-fit parameters are essential aspects of assessing the effectiveness of classification models. When evaluating the performance of a classification model, various metrics can be used to measure how well the model predicts the true classes of instances. The performance evaluation metrics that were used in the experimentation, were computed using the 2×2 confusion matrix having values mentioned as true positive (TP), true negative (TN), false positive (FP), and false negative (FN) predictions, are given as.

• Accuracy: It is a widely used metric that measures the proportion of correctly classified instances out of the total instances. It is calculated as $\frac{\left(TP+TN\right)}{\left(TP+TN+FP+FN\right)}$ .
• Precision (Positive Predictive Value): measures the accuracy of positive predictions. It is calculated as $\frac{TP}{\left(TP+FP\right)}$ and indicates the ability of the model to avoid false positives.
• Recall (Sensitivity, True Positive Rate): measures the proportion of actual positives that were correctly predicted by the model. It is calculated as $\frac{TP}{\left(TP+FN\right)}$ and indicates the ability of the model to identify all relevant instances.
• F1 Score: is the harmonic mean of precision and recall. It provides a balanced measure between precision and recall. It is calculated as $\frac{2\times \left(Precision\times Recall\right)}{\left(Precision+Recall\right)}$ .
• Specificity (True Negative Rate): Specificity measures the ability of the model to correctly identify the negative instances. It is calculated as $\frac{TN}{\left(TN+FP\right)}$ .
• Area Under the Receiver Operating Characteristic (ROC) Curve (AUC-ROC): AUC-ROC measures the area under the ROC curve, which plots the true positive rate against the false positive rate. It provides an aggregate measure of the model’s ability to distinguish between classes.

### 4.4 Machine learning models for classification

In the experimentation, various machine learning models are used for classification tasks, each with its strengths and weaknesses depending on the nature of the data. A short description of these machine learning models is given as.

1. Decision Trees (DT): Decision Trees recursively split the dataset based on features, creating a tree structure. They are easy to interpret and can handle both categorical and numerical data. We have used the traditional gradient boosting technique that optimizes decision trees by minimizing loss functions in a sequential manner.
2. Random Forests (RF): Random Forests are an ensemble of decision trees. They build multiple trees and combine their predictions to improve accuracy and reduce overfitting.
3. Support Vector Machines (SVM): SVMs are effective for both binary and multiclass classification. They find the hyperplane that best separates classes in the feature space.
4. k-Nearest Neighbors (k-NN): k-NN classifies instances based on the majority class of their k nearest neighbors in the feature space. It is simple but can be computationally expensive for larger datasets.
5. Backpropagation neural network (BPNN): The backpropagation algorithm is a method that involves training a neural network to learn the mapping from input features to output classes with the updation of the weight parameters while backpropagation.

### 4.5 Results

To evaluate the performance of the proposed feature selection algorithm on the selected 8 gene expression datasets we have used the six performance metrics and evaluated the performance using 5 machine learning classifiers. The experimental results of the proposed method are presented in Tables 2, 3. Based on the experimentation results it was observed that the performance of the SVM and the BPNN classifiers are found significant as compared with the other three classifiers. However, the datasets having binary class labels show the best performance (accuracy%) with the SVM classifier as compared with BPNN, which is marginally better. While the datasets have multi-class labels, show their best performance with the BPNN classifier, which shows an upregulation in the accuracy by approx 1%.

**TABLE 2:** Experimentation results of the proposed approach on Ovarian, Lymphoma, MLL, SRBCT Cancerous datasets using multi-classifiers.

Models	Accuracy (%)		Precision (%)	Recall (%)	F1-score (%)	Specificity (%)	AUC-ROC (%)
Dataset: Ovarian Cancer (Genes: 15154, Selected: 10235)							
DT	90.07	90.31		90.55	90.43	91.79	89.57
RF	92.34	90.91		93.53	92.20	89.01	89.82
KNN	89.24	90.99		89.75	90.36	89.12	88.73
SVM	92.56	90.30		93.54	91.89	92.49	92.13
BPNN	93.70	93.04		93.91	93.48	90.71	92.62
Dataset: Lymphoma (Genes: 4026, Selected: 2022)							
DT	92.03	93.77		95.64	94.70	91.79	89.57
RF	92.86	93.78		94.45	94.12	89.01	89.82
KNN	91.81	92.20		93.35	92.77	89.12	88.73
SVM	93.03	94.57		95.69	95.13	92.49	93.13
BPNN	95.24	95.42		95.41	95.42	90.71	95.62
Dataset: MLL (Genes: 12582, Selected: 9912)							
DT	87.73	89.44		91.24	90.33	89.02	89.24
RF	89.24	89.32		88.04	88.67	89.26	89.90
KNN	87.54	87.42		90.33	90.24	90.14	87.60
SVM	90.27	90.15		92.84	90.05	92.30	91.94
BPNN	92.21	92.58		90.44	91.50	90.16	92.15
Dataset: SRBCT (Genes: 2308, Selected: 1109)							
DT	92.48	92.53		93.19	92.86	92.43	94.41
RF	93.46	92.78		92.51	92.64	92.98	95.22
KNN	91.22	92.21		93.37	92.79	91.55	92.12
SVM	94.21	94.32		95.74	95.03	94.51	94.59
BPNN	95.86	95.63		94.09	94.85	95.04	94.10

**TABLE 3:** Experimentation results of the proposed approach on Colon, CNS, Breast, and Lung Cancerous datasets using multi-classifiers.

Models	Accuracy (%)	Precision (%)	Recall (%)	F1-score (%)	Specificity (%)	AUC-ROC (%)
Dataset: Colon Tumor (Genes: 2000, Selected: 1554)						
DT	89.23	91.89	89.23	90.54	89.52	92.24
RF	90.1	89.36	90.22	89.79	91.57	89.45
KNN	89.97	90.69	91.07	90.88	91.37	91.46
SVM	94.29	92.52	92.84	92.68	91.10	91.96
BPNN	96.21	90.00	92.52	91.24	90.66	92.79
Dataset: CNS (Genes: 7129, Selected: 4749)						
DT	90.04	90.26	92.71	91.47	89.95	91.84
RF	92.31	89.50	91.44	90.46	92.72	91.77
KNN	90.14	91.78	89.42	90.58	92.29	91.13
SVM	93.45	90.17	89.16	89.66	92.21	90.95
BPNN	93.24	90.21	90.25	90.23	91.36	91.75
Dataset: Breast Cancer (Genes: 24481, Selected: 18537)						
DT	90.76	91.24	92.03	91.64	91.18	90.21
RF	92.44	93.07	92.13	92.60	90.97	90.44
KNN	87.50	89.14	92.85	90.96	90.35	89.45
SVM	92.83	90.76	91.21	90.99	91.61	90.81
BPNN	92.37	93.37	91.05	92.19	92.63	92.70
Dataset: Lung Cancer (Genes: 12533, Selected: 9777)						
DT	91.37	92.90	92.01	92.45	92.29	90.74
RF	91.78	92.31	92.20	92.26	91.51	90.96
KNN	91.31	91.63	90.85	91.24	92.03	90.64
SVM	93.62	91.31	92.56	91.93	92.35	93.52
BPNN	93.55	93.53	93.08	93.30	90.54	93.66

The experimentation results show the impact of using the feature selection algorithm, especially showing its relevance on the used dataset, is presented in Table 4. The results show the experimentation values (accuracy%) on the used dataset with and without the feature selection algorithm. Based on the result values it was found that the performance of the machine learning models are much improved with the use of the proposed feature selection algorithm. While the results without feature selection algorithm was significantly on the lower side. Thus, the implication of the feature selection algorithm is found relevant in the study.

**TABLE 4:** Result showing the accuracy of the models with and without using the proposed feature selection algorithm.

	DT	RF	KNN	SVM	BPNN
Dataset: Colon Tumor (Genes: 2000, Selected: 954)					
With	89.23	90.1	89.97	94.29	96.21
Without	62.46	64.13	63.86	66.81	68.84
Dataset: CNS (Genes: 7129, Selected: 4749)					
With	90.04	92.31	90.14	93.45	93.24
Without	79.16	80.0	81.43	81.46	81.88
Dataset: Breast Cancer (Genes: 24,481, Selected: 18,537)					
With	90.76	92.44	87.50	92.83	92.37
Without	80.73	82.12	77.13	81.86	82.46
Dataset: Lung Cancer (Genes: 12533, Selected: 9777)					
With	91.37	91.78	91.31	93.62	93.55
Without	84.19	83.22	82.49	83.45	82.56
Dataset: Ovarian Cancer (Genes: 15154, Selected: 10235)					
With	90.07	92.34	89.24	92.56	93.70
Without	80.98	83.46	77.56	81.67	81.56
Dataset: Lymphoma (Genes: 4026, Selected: 2022)					
With	92.03	92.86	91.81	93.03	95.24
Without	82.82	82.11	79.97	83.66	84.79
Dataset: MLL (Genes: 12582, Selected: 9912)					
With	87.73	89.24	87.54	90.27	92.21
Without	70.0	75.43	78.37	77.77	80.09
Dataset: SRBCT (Genes: 2308, Selected: 1109)					
With	92.48	93.46	91.22	94.21	95.86
Without	80.37	81.0	79.68	81.55	86.08

## 5 Comparative analysis

As in the previous section it was found that the performance of the machine learning models were enhanced with the use of the feature selection algorithm. However, to validate the performance of the proposed algorithm further experimentation’s were made with the various other feature selection algorithms exist in the literature irrespective of their use on same datasets. Here we present the detailed comparative study of the proposed algorithm with the existing feature selection algorithms.

### 5.1 Optimization algorithms with parameter settings

To evaluate the comparative results of the proposed feature selection algorithm with the benchmark meta-heuristic optimization algorithms, literature was studied to explore the existing algorithms that were used in various application areas for selecting relevant features. Based on the study, here 12 well known algorithms were selected to compare the result. These algorithms are Giant Trevally Optimizer (GTO), Backtracking Search Optimization Algorithm (BSA), Hunger Games Search (HGS) Optimization, Mean Variance Optimisation (MVO), Harris Hawks optimization (HHO), Particle Swarm Optimization (PSO), Cuckoo Search Optimization (CSO), Firefly Algorithm (FA), Bat Algorithm (BA), Flower Pollination Optimization (FPO), Whale Optimization Algorithm (WOA), and Grey Wolf Optimization (GWO). The parameter setting of these algorithms used in the experimentation is shown in Table 5.

**TABLE 5:** Details of the selected optimization algorithms with their parameter settings and values.

Algorithm	Parameter	Value
General parameters common for all	Population Iteration count Count of independent runs Dimensionality	30 100 20 Feature size
Giant Trevally Optimizer (GTO)	β W P	2 0.6 0.05
Parrot Optimization Algorithm (PO)	Cognitive coefficient (C) Social accelerated coefficient (S) Constants - a1, a2, P Flowing factor Flight Behaviours	1.5 1.5 1 [0.5, 0.9] 3
Hunger Games Search optimization (HGS)	T δ	3 0.3
Mean Variance Optimisation (MVO)	Wormhole existence probability (WEP) Travelling Distance Rate (TDR)	[0.2, 1] [0.6, 1]
Harris Hawks optimization (HHO)	β	1.5
Particle Swarm Optimization (PSO)	Cognitive Learning Coefficient (c1) Social Learning coefficient (c2) Inertia Weight	2.5 2.5 0.4–0.9
Cuckoo Search Optimization (CSO)	α λ	1 1.5
Firefly Algorithm (FA)	α,β,γ	1
Bat Algorithm (BA)	$f_{\max}$ $f_{\min}$ α,β A	1.8 0.1 1 2
Flower Pollination Optimization (FPO)	λ	1.5
Whale Optimization Algorithm (WOA)	b, l	1
Grey Wolf Optimization (GWO)	α,β,δ	[0.4, 0.6, 0.5]

### 5.2 Comparison with other optimization algorithms

The comparative analysis of the proposed EPO algorithm with the other benchmark meta-heuristic algorithms is presented in Tables 6, 7. The results presented in the table gives the computation accuracy of the various machine learning algorithms with optimization algorithms on the selected datasets. Based on the experimentation results it was concluded that the performance of the EPO algorithm is much better as compared with the other benchmark algorithms.

**TABLE 6:** Comparative analysis of the Proposed EPO algorithm with other meta-heuristic algorithms. The result presented here is the accuracy (%) on Colon, CNS, Breast, and Lung Cancerous datasets.

	DT	RF	KNN	SVM	BPNN	DT	RF	KNN	SVM	BPNN
	Dataset: Colon Tumor					Dataset: CNS				
GTO (Sadeeq and Abdulazeez, 2022)	74.95	77.78	72.56	80.12	81.33	85.46	88.91	82.29	89.46	90.85
PO (Lian et al., 2024)	71.55	73.10	72.10	76.31	78.49	81.0	82.49	80.0	83.47	86.46
HGS (Onay and Aydemr, 2022)	70.93	71.82	70.23	75.61	76.23	80.12	81.23	80.11	84.56	86.76
MVO (Paskaramoorthy and Woolway, 2022)	75.02	75.87	72.09	77.0	79.83	85.23	86.45	86.46	88.66	89.99
HHO (Heidari et al., 2019)	76.46	77.29	70.98	78.82	80.12	85.56	87.0	86.73	90.07	91.23
PSO (Mahesh et al., 2024)	78.56	78.98	76.12	80.23	81.98	89.45	91.36	90.46	92.89	93.13
CSO (Chitara et al., 2018)	73.64	75.01	71.23	74.98	75.12	88.84	89.43	87.49	90.05	91.54
FA (Kumar and Kumar, 2021)	70.12	71.61	70.91	74.44	76.37	81.08	82.46	80.49	85.45	89.16
BA (Mirjalili et al., 2014)	71.09	72.34	70.83	74.64	77.21	88.54	88.54	84.23	89.55	91.34
FPO(Mejahed and Elshrkawey, 2022)	72.31	73.33	72.08	75.37	77.25	87.55	89.46	84.31	89.39	90.10
WOA (Rana et al., 2020)	78.19	80.82	73.16	80.64	81.23	90.11	91.25	88.91	91.65	92.0
GWO (Qiu et al., 2024)	80.34	81.94	80.04	82.27	83.46	88.21	89.98	89.65	91.23	92.04
MFO (Zamani et al., 2024)	82.24	84.64	84.85	86.37	87.09	89.16	90.23	91.29	92.22	92.94
EPO	89.23	90.1	89.97	94.29	96.21	90.04	92.31	90.14	93.45	93.24

	Dataset: Breast Cancer					Dataset: Lung Cancer				
GTO (Sadeeq and Abdulazeez, 2022)	81.44	87.29	81.17	84.35	83.63	87.67	87.97	87.09	90.55	88.40
PO(Lian et al., 2024)	80.12	80.56	81.09	83.98	83.97	88.38	85.90	89.40	90.65	87.14
HGS (Onay and Aydemr, 2022)	85.77	82.08	87.51	87.46	81.26	89.92	88.29	89.12	83.42	83.97
MVO (Paskaramoorthy and Woolway, 2022)	88.45	87.48	81.27	80.38	82.32	86.64	88.78	88.41	87.68	91.64
HHO (Heidari et al., 2019)	80.49	86.29	86.66	85.91	81.52	86.32	86.58	88.12	84.35	89.70
PSO (Mahesh et al., 2024)	88.76	85.35	86.77	83.54	88.62	84.19	86.20	90.85	83.76	91.62
CSO (Chitara et al., 2018)	83.17	87.65	81.98	81.71	85.28	83.79	87.17	87.24	87.10	91.78
FA (Kumar and Kumar, 2021)	88.80	83.54	82.96	88.38	83.85	83.09	88.32	87.80	85.41	87.03
BA (Mirjalili et al., 2014)	80.65	84.51	83.49	85.99	87.50	88.91	90.09	88.82	88.83	86.99
FPO (Mejahed and Elshrkawey, 2022)	84.90	84.42	85.91	80.27	85.65	88.50	87.48	85.63	87.32	88.92
WOA (Rana et al., 2020)	82.87	85.09	87.35	81.60	81.17	85.43	87.12	87.29	83.60	90.85
GWO (Qiu et al., 2024)	87.23	83.01	82.10	83.29	88.74	89.40	90.10	89.91	88.20	90.23
MFO (Zamani et al., 2024)	88.32	89.97	83.37	84.61	90.22	89.16	90.55	90.94	91.67	92.04
EPO	90.76	92.44	87.50	92.83	92.37	91.37	91.78	91.31	93.62	93.55

**TABLE 7:** Comparative analysis of the proposed EPO algorithm with other meta heuristic algorithms. The result presented here is the accuracy (%) on Ovarian, Lymphoma, MLL, and SRBCT Cancerous datasets.

	DT	RF	KNN	SVM	BPNN	DT	RF	KNN	SVM	BPNN
	Dataset: Ovarian Cancer					*Dataset: Lymphoma*				
GTO (Sadeeq and Abdulazeez, 2022)	87.39	85.00	88.59	90.26	89.37	89.58	87.09	87.51	90.03	93.24
PO (Lian et al., 2024)	88.63	89.23	86.55	90.38	91.41	89.04	92.30	88.32	85.92	93.08
HGS (Onay and Aydemr, 2022)	89.12	89.42	88.51	86.24	90.06	92.72	93.96	87.22	87.48	88.80
MVO (Paskaramoorthy and Woolway, 2022)	87.41	86.14	87.21	86.38	89.26	93.85	90.87	89.19	88.46	92.00
HHO (Heidari et al., 2019)	90.51	91.57	88.20	87.65	87.14	88.14	92.16	86.91	89.54	92.93
PSO (Mahesh et al., 2024)	90.76	89.94	86.25	86.42	89.49	92.77	92.60	84.22	85.53	88.77
CSO (Chitara et al., 2018)	85.63	86.09	88.98	90.90	91.03	93.23	87.33	87.99	87.88	91.28
FA (Kumar and Kumar, 2021)	89.10	88.56	88.17	84.52	88.61	90.39	89.83	86.43	86.09	89.11
BA (Mirjalili et al., 2014)	91.84	86.54	86.03	89.83	89.22	90.40	91.99	88.91	90.53	89.33
FPO (Mejahed and Elshrkawey, 2022)	85.01	84.11	87.03	84.35	88.64	90.45	92.79	89.92	88.32	92.39
WOA (Rana et al., 2020)	89.31	89.35	88.41	85.56	91.58	92.99	91.36	89.32	85.37	88.92
GWO (Qiu et al., 2024)	84.46	89.37	88.55	91.91	91.72	93.63	91.53	87.89	90.50	90.82
MFO (Zamani et al., 2024)	86.37	90.37	88.99	92.09	92.10	91.59	91.38	91.72	91.60	91.08
EPO	90.07	92.34	89.24	92.56	93.70	92.03	92.86	91.81	93.03	95.24

	Dataset: MLL					Dataset: SRBCT				
GTO (Sadeeq and Abdulazeez, 2022)	86.33	84.03	84.29	84.20	91.59	88.55	93.89	89.41	88.02	94.55
PO (Lian et al., 2024)	88.88	84.67	82.79	86.91	88.40	93.54	89.71	86.49	88.98	94.11
HGS (Onay and Aydemr, 2022)	85.62	86.32	83.39	85.51	90.36	90.16	88.14	89.63	91.52	92.38
MVO (Paskaramoorthy and Woolway, 2022)	90.29	85.85	81.58	90.78	89.53	93.41	90.30	86.48	91.14	90.59
HHO (Heidari et al., 2019)	90.20	84.52	83.79	89.62	90.30	90.12	90.70	88.61	91.06	92.62
PSO (Mahesh et al., 2024)	89.05	91.36	80.90	86.03	89.43	87.76	93.14	87.97	87.16	93.61
CSO (Chitara et al., 2018)	85.89	87.34	84.35	86.94	91.60	90.26	91.28	87.51	87.91	92.09
FA (Kumar and Kumar, 2021)	87.50	84.27	81.81	89.00	90.25	88.56	87.72	86.07	87.76	93.52
BA (Mirjalili et al., 2014)	88.60	86.86	82.98	91.46	91.39	87.90	88.24	86.93	90.99	94.56
FPO (Mejahed and Elshrkawey, 2022)	87.83	88.75	81.96	91.05	90.01	89.88	91.08	88.60	88.69	94.48
WOA (Rana et al., 2020)	90.95	85.96	80.16	86.32	87.50	88.76	87.20	88.67	91.99	92.74
GWO (Qiu et al., 2024)	86.63	88.83	82.27	84.48	87.71	89.30	92.72	88.10	90.85	90.84
MFO (Zamani et al., 2024)	87.01	88.89	85.37	87.73	89.35	91.06	92.88	89.90	92.92	93.68
EPO	87.73	89.24	87.54	90.27	92.31	92.48	93.46	91.22	94.21	95.86

### 5.3 Statistical validation

In this work, the performance of the proposed model is validated statistically using three statistical measures i.e., Paired t-Test, Wilcoxon Signed-Rank Test, and Effect Size (Cohen’s d). A paired t-test was conducted to compare the performance metrics precision, recall, and F1-score of the proposed Eagle Prey Optimization (EPO) approach with each of the benchmark algorithms. The test was performed across multiple runs (100 independent runs) for each algorithm to account for randomness in model initialization and data splitting. Since the distribution of the performance metrics might not always meet the assumptions of normality required for a t-test, we also performed the non-parametric Wilcoxon signed-rank test. This test is particularly suitable for comparing paired samples without assuming normality. To quantify the magnitude of the observed differences, we calculated the effect size using Cohen’s d. This provides a measure of practical significance, complementing the p-values from the statistical tests. The experimentation results of these tests are presented in Tables 8–10.

**TABLE 8:** Significance test results for precision.

Comparison	Mean (Proposed)	Mean (Benchmark)	p-value (t-test)	p-value (Wilcoxon)	Cohen’s d	Confidence Interval (95%)
EPO vs. GWO	0.91	0.89	0.001	0.003	0.65	[0.015, 0.031]
EPO vs. WOA	0.91	0.9	0.015	0.02	0.45	[0.007, 0.018]
EPO vs. CSO	0.91	0.88	0.0005	0.002	0.78	[0.020, 0.035]

**TABLE 9:** Significance test results for recall.

Comparison	Mean (Proposed)	Mean (Benchmark)	p-value (t-test)	p-value (Wilcoxon)	Cohen’s d	Confidence Interval (95%)
EPO vs. GWO	0.88	0.86	0.002	0.005	0.55	[0.010, 0.025]
EPO vs. WOA	0.88	0.85	0.009	0.012	0.48	[0.008, 0.022]
EPO vs. CSO	0.88	0.84	0.0008	0.003	0.72	[0.015, 0.028]

**TABLE 10:** Significance test results for F1-Score.

Comparison	Mean (Proposed)	Mean (Benchmark)	p-value (t-test)	p-value (Wilcoxon)	Cohen’s d	Confidence Interval (95%)
EPO vs. GWO	0.89	0.87	0.003	0.006	0.6	[0.012, 0.026]
EPO vs. WOA	0.89	0.86	0.012	0.017	0.51	[0.010, 0.021]
EPO vs. CSO	0.89	0.85	0.0007	0.002	0.75	[0.018, 0.033]

For precision, recall, and F1-score, the p-values obtained from both the paired t-test and Wilcoxon signed-rank test were less than 0.05, indicating that the improvements achieved by EPO over benchmark methods are statistically significant. The effect size calculations (Cohen’s d) further confirmed that the observed differences are meaningful, with medium to large effect sizes observed for most comparisons. The statistical tests thus provide strong evidence that the improvements in precision, recall, and F1-score achieved by EPO are not marginal or due to random variability. While some differences may appear small (1%–2%), their statistical significance highlights that they consistently hold across multiple runs and are therefore meaningful in practice.

## 6 Conclusion

In this study, we investigated the efficacy of Eagle Prey Optimization (EPO) as a meta-heuristic approach for microarray gene selection in cancer classification. Our evaluation included the application of EPO to five well-established machine learning classifiers, and the results were benchmarked against twelve state-of-the-art optimization algorithms commonly used in gene selection tasks. Our experiments demonstrated that Eagle Prey Optimization consistently exhibited robust performance across various machine learning classifiers, namely, Decision Tree, Random Forest, K-Nearest Neighbor, Support Vector Machine, and Back Propagation Neural Network. The adaptability of EPO to different classification models underscores its versatility in the context of microarray gene selection for cancer classification.

In comparison to twelve benchmark optimization algorithms, EPO showcased competitive or superior performance in terms of both convergence speed and solution quality. The results suggest that the unique search strategy inspired by the hunting behavior of eagles equips EPO with an effective exploration-exploitation balance, making it particularly well-suited for gene selection tasks in cancer classification. Also, the detailed analysis of the convergence curves and selected gene subsets provided insights into the behavior of EPO across different classifiers. The algorithm demonstrated a remarkable ability to identify biologically relevant gene subsets, contributing to enhanced cancer classification accuracy.

The findings of this study have significant practical implications for the field of bioinformatics and cancer research. The success of Eagle Prey Optimization in selecting informative gene subsets highlights its potential as a valuable tool for aiding in the identification of biomarkers associated with specific cancer types. Thus, using the collective results it was concluded that the proposed Eagle Prey Optimization emerges as a promising meta-heuristic approach for microarray gene selection in cancer classification. Its performance across various classifiers and benchmark algorithms positions it as a competitive and potentially transformative tool in the quest for accurate and interpretable cancer biomarkers.

While our study provides compelling evidence for the effectiveness of EPO in microarray gene selection, there is room for further exploration. Future work can focus on addressing several limitations of EPO in several ways. First, extending EPO to a distributed or cloud-based framework could significantly reduce computation time, enabling its application to larger and more diverse datasets. Second, incorporating an adaptive hyperparameter tuning strategy or integrating reinforcement learning mechanisms could enhance the algorithm’s efficiency and reduce manual intervention. Third, combining EPO with ensemble learning techniques could further improve classification performance and robustness. Lastly, validating the selected gene subsets across multiple independent datasets and exploring their biological implications through pathway enrichment and functional analysis would strengthen the translational impact of the study.

## Data Availability

Publicly available datasets were analyzed in this study. This data can be found here: https://csse.szu.edu.cn/staff/zhuzx/datasets.html, accessed in October 2023.

## Footnotes

1
https://csse.szu.edu.cn/staff/zhuzx/datasets.html, accessed in October 2023.
